# Keratin 8/18 Regulation of Cell Stiffness-Extracellular Matrix Interplay through Modulation of Rho-Mediated Actin Cytoskeleton Dynamics

**DOI:** 10.1371/journal.pone.0038780

**Published:** 2012-06-07

**Authors:** François Bordeleau, Marie-Eve Myrand Lapierre, Yunlong Sheng, Normand Marceau

**Affiliations:** 1 Centre de recherche en cancérologie and Centre de Recherche du Centre hospitalier de Québec, Quebec City, Quebec, Canada; 2 Centre d'optique photonique et laser, Université Laval, Quebec City, Quebec, Canada; Emory University/Georgia Insititute of Technology, United States of America

## Abstract

Cell mechanical activity generated from the interplay between the extracellular matrix (ECM) and the actin cytoskeleton is essential for the regulation of cell adhesion, spreading and migration during normal and cancer development. Keratins are the intermediate filament (IF) proteins of epithelial cells, expressed as pairs in a lineage/differentiation manner. Hepatic epithelial cell IFs are made solely of keratins 8/18 (K8/K18), hallmarks of all simple epithelia. Notably, our recent work on these epithelial cells has revealed a key regulatory function for K8/K18 IFs in adhesion/migration, through modulation of integrin interactions with ECM, actin adaptors and signaling molecules at focal adhesions. Here, using K8-knockdown rat H4 hepatoma cells and their K8/K18-containing counterparts seeded on fibronectin-coated substrata of different rigidities, we show that the K8/K18 IF-lacking cells lose their ability to spread and exhibit an altered actin fiber organization, upon seeding on a low-rigidity substratum. We also demonstrate a concomitant reduction in local cell stiffness at focal adhesions generated by fibronectin-coated microbeads attached to the dorsal cell surface. In addition, we find that this K8/K18 IF modulation of cell stiffness and actin fiber organization occurs through RhoA-ROCK signaling. Together, the results uncover a K8/K18 IF contribution to the cell stiffness-ECM rigidity interplay through a modulation of Rho-dependent actin organization and dynamics in simple epithelial cells.

## Introduction

The ability of cells to sense and adapt to mechanical cues from the extracellular matrix (ECM) is crucial for several biological processes, including the involvement of mechanical force in dictating embryonic development [1]. For instance, embryonic stem cells progressively stiffen as they undergo differentiation, and tune their stiffness to the rigidity variation of the underlying ECM [2]. In a similar way, there is compelling evidence for the involvement of increased ECM rigidity in promoting the emergence of primary tumors and the subsequent metastatic migration of escaping cells [3]. Moreover, aggressive tumorigenic cells in suspension, where they are independent from ECM interaction, are more compliant than less aggressive cells, which in turn are more compliant than healthy cells [4]; still, tumorigenic cells seeded on a rigid ECM substratum exhibit increased contractility [5]. Such differences in cell behavior, in link with changes in ECM rigidity, highlight how important it is for cells to adapt to mechanical cues, in order to counterbalance ECM constraints.

An ECM-derived stress is perceived and integrated intracellularly through the participation of integrin receptors, acting as mechanotransducers interfacing with signaling cascades and actin cytoskeleton at focal adhesions (FAs) to elicit cellular responses, such as cell migration and contractility [1], [6]. Experimentally, cell contractility and its associated internal stiffness can be assessed by measuring the force-induced displacement of fibronectin (FN)-coated beads attached at FAs generated at the dorsal cell surface [7], [8]. Such measurements at the cellular level have established, for instance, that a de-polymerization of the actin cytoskeleton reduces cell stiffness, recognizing this cytoskeletal network as a prominent contributor of the cellular response to mechanical force applied at FAs [8], [9]. At the molecular level, the balance between internal stiffness and extracellular force exerted at FAs is maintained by modulating the fibrillar actin contractility [5], [6], [10], which occurs through activation of Rho and the effector ROCK, a regulator of the myosin light chain [11], [12]. Such cell-generated Rho-dependent contractility points to a prominent actin cytoskeleton involvement in the interplay between cell stiffness and ECM rigidity.

Keratins (Ks), the intermediate filament (IF) proteins of epithelial cells, constitute the largest family of cytoskeletal proteins and are grouped into type I (K9–28) and type II (K1–K8 and K71–K80) subfamilies [13]. Keratin IFs are obligate heteropolymers that include at least one type I and one type II keratin, and are coordinately expressed as specific pairs in a cell lineage and differentiation manner. IFs from all simple epithelial cells contain K8/K18 and most possess 2–3 other keratins as well [14], [15]. Notably, K8 and K18 are the ancestral genes for the multiple specialized Type II and Type I keratin classes, respectively, and constitute the first cytoplasmic IF genes expressed in the embryo, at the time of stem cell differentiation along the different cell lineages [16], [17]. With regard to cancer, there is accumulating evidence showing, for instance, that persistence of K8/K18 IFs is a hallmark of invasive squamous cell carcinoma, where such perturbed K8/K18 expression appears to contribute to cell invasiveness through an actin-dependent motility [18]. In addition, point mutations in K8 and K18 genes lead to IF disorganization and predispose to liver cirrhosis [19] and in turn, cirrhosis reflects increasing hepatic tissue stiffness, a ECM-linked mechanical alteration often associated with the emergence of hepatocellular carcinoma [20], [21]. Thus, considering that keratin IFs constitute a resilient yet flexible cytoskeletal network that is largely responsible for the capacity of epithelial cells to sustain mechanical stress [17], one can hypothesize that K8/K18 IFs need to be included in the interplay that takes place between actin-mediated cell stiffness and ECM rigidity in simple epithelial cells.

In the work reported here, we addressed this hypothesis using monolayer cultures of K8-knockdown H4-II-E-C3 (shK8b) rat hepatoma cells and their K8/K18-containing counterparts (H4ev) [22]. Of note, the IFs of these hepatic cells, like those of hepatocytes, are made up of K8/K18 only [15], [23], which means that a loss of K8 leads to the degradation of K18, and thus provides simple epithelial cells lacking K8/K18 IFs [24]. H4ev cells and shK8b cells were seeded on FN-coated substrata of different rigidities, as described before [25]; a laser tweezers-mediated force was applied on a FN-coated microbead attached to integrins [9], [26]; and the cell stiffness at FAs was evaluated by measuring the bead displacement [9]. In addition, we assessed the involvement of the Rho-ROCK in connection with shK8b cell versus H4ev cell stiffness and fibrillar actin organization. The results uncover a strategic intervention of K8/K18 IFs in the cell stiffness-ECM rigidity interplay through a modulation of Rho-dependent actin organization and dynamics in simple epithelial cells.

## Results

### Low rigidity substratum differentially affects H4ev and shK8b cell shape

**Figure 1:**
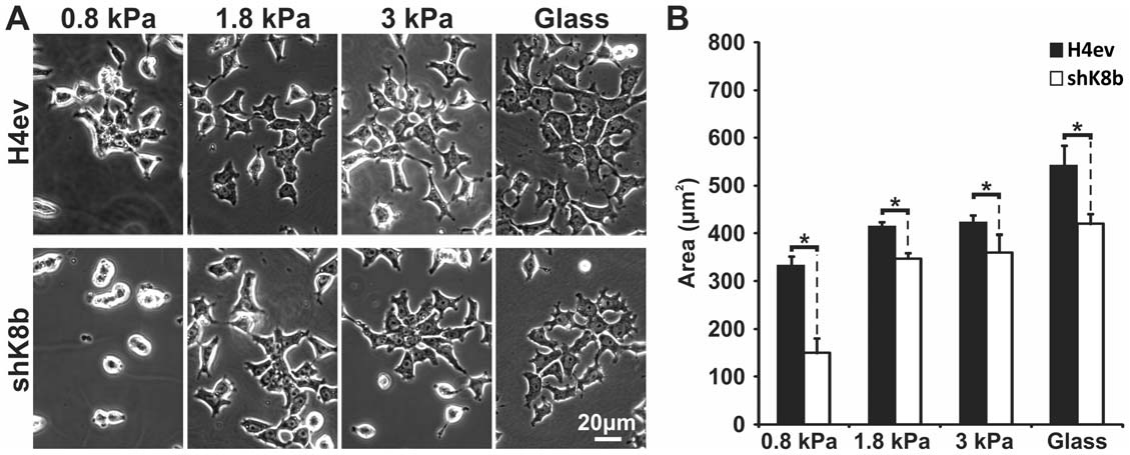
Substratum rigidity differentially affects H4ev and shK8b cell shapes. (A) Phase contrast images of cells one day after seeding on FN-gels of increasing rigidity or in FN-coated dishes, showing a more shK8b round cell shape under low FN-gel rigidity compared to the H4ev spread shape. For higher gel rigidity, both H4ev and shK8b cells show comparable spread shapes. (B) Measurements of H4ev and shK8b cell areas from the corresponding seeding conditions. N = 60. Bars denote SE. *, p<0.05 for H4ev versus shK8b.

Since the shape of adherent cells largely depends on an interplay between cytoskeleton and ECM [27], we first assessed to which extend a loss of the K8/K18 IF network in hepatic cells affects their shape in response to FN-gel rigidities that are representative of normal and fibrotic hepatic tissue [28], [29], [30]. H4ev and shK8b cells were seeded at an intermediate density on FN-gel of 0.8, 1.8 and 3 kPa or on FN-coated glass (>3 GPa). As shown in Fig. 1A, the shape of H4ev cells at day-1 post-seeding appeared similar over the whole range of FN-gel rigidity. In contrast, shK8b cells seeded on a 0.8 kPa gel maintained a round shape despite their firm adhesion on the gel, even for those cells that maintained cell-cell contacts, whereas their shape was similar to that of H4ev cells on a 1.8 kPa or 3 kPa FN-gel (Fig. 1A). In fact, cell area measurements revealed that the spreading ability of shK8b cells remained below that of H4ev cells over the whole range of FN-gel rigidity (Fig. 1B). Of note, shK8b cells plated on 0.8 kPa FN-gels exhibit a size equivalent to that we observed previously for the same cell type held in suspension [24]. It is also worth noticing that the present finding using the FN-coated glass substratum confirmed our previous data using the same seeding condition [22], [24]. At day-3, H4ev cells formed confluent monolayers on all gels. This was not the case for shK8b cells seeded on a 0.8 kPa FN-gel, in which case the cells maintained a rounded shape, even when plated at higher density (data not shown). Together, these observations readily pointed to an impaired cell mechanosensing as a result of the K8/K18 IF loss.

### Differential actin organization in H4ev and shK8b cells in response to substratum rigidity

**Figure 2:**
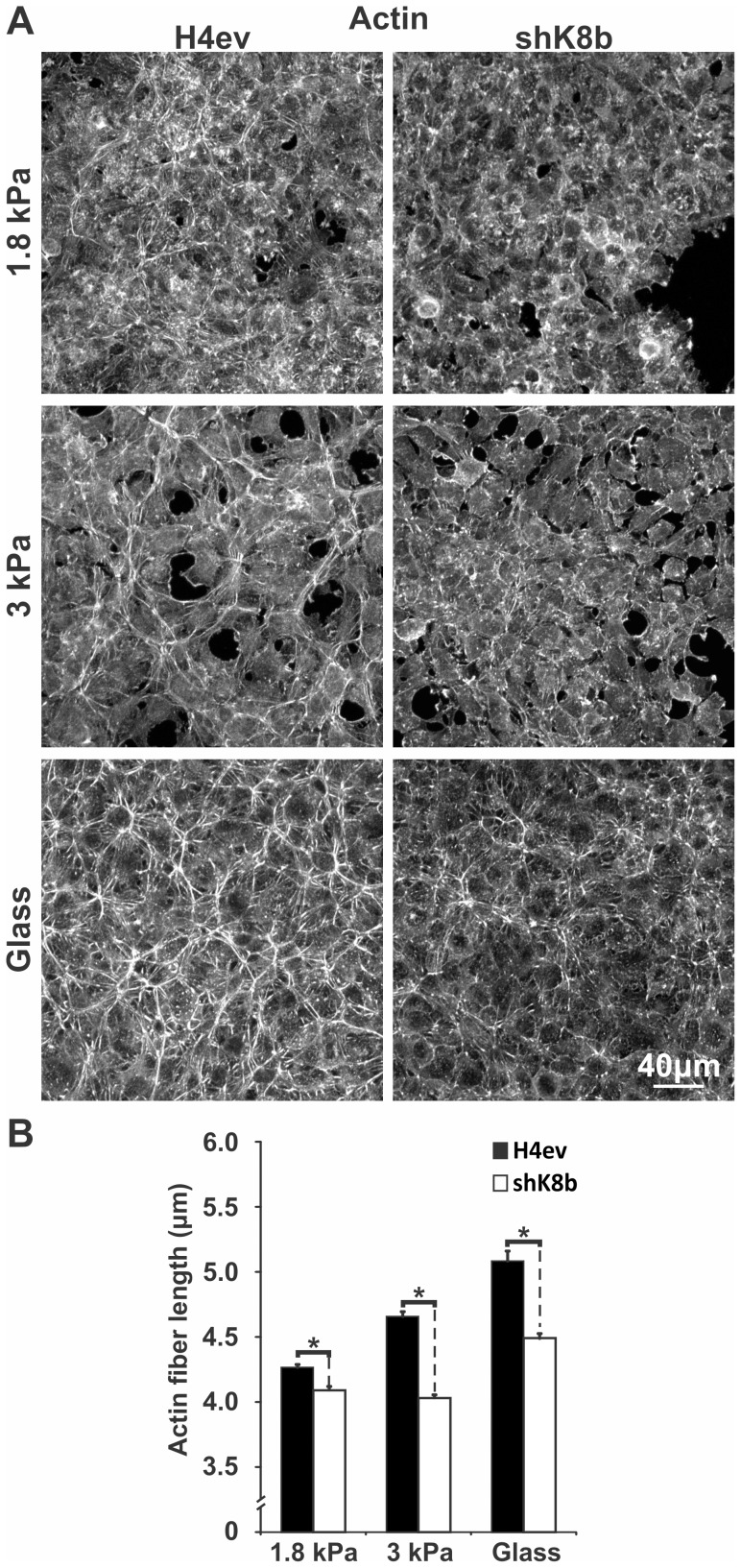
Actin fiber organization in H4ev and shK8b cells seeded on substratum of increasing rigidity. (A) Maximum projection confocal images of fibrillar actin in 3-day monolayers of cells seeded on FN-gels of increasing rigidity or in FN-coated dishes, showing distinct actin organizations in H4ev versus shK8b monolayers. In both cell monolayers, the number and density of actin fibers increase with substratum rigidity. (B) Average actin fiber length estimates from the corresponding seeding conditions, showing shorter actin fibers in shK8b compared to those in H4ev cells. Bars denote SE. *, p<0.05 for H4ev versus shK8b.

Considering that a change in ECM rigidity modulates fibrillar actin organization in adherent cells [27], [31], we determined here the effect of the K8/K18 IF loss on actin organization in hepatic cells at day-3 post-seeding on FN-gels. H4ev cells seeded on a 1.8 kPa FN-gel showed numerous actin fibers, but did not form a coherent fiber network, which contrasted with the interconnected network formed on 3 kPa FN-gel; it was even more so for H4ev cells on FN-coated glass, where the actin fibers formed a compact network across the monolayer (Fig. 2A). No such fibrillar actin networks formed in shK8b cells seeded on 1.8 and 3 kPa FN-gel but still, seeding on FN-coated glass yielded networks of non-interconnected actin fibers. Quantitative estimates revealed a substrate rigidity-dependent increase in actin fiber length in H4ev cells (Fig. 2B). In contrast, in shK8b cells, the actin fiber length did not increase between the 1.8 kPa and 3 kPa FN-gels, and even though the fiber length increased for cells seeded on FN-coated glass, the actin fibers remained shorter when compared to those in H4ev cells (Fig. 2B). These results demonstrate that K8/K18 IFs constitute a modulator of actin fiber organization over a wide range of ECM rigidity.

### FN modulation of H4ev versus shK8b cell stiffness

**Figure 3:**
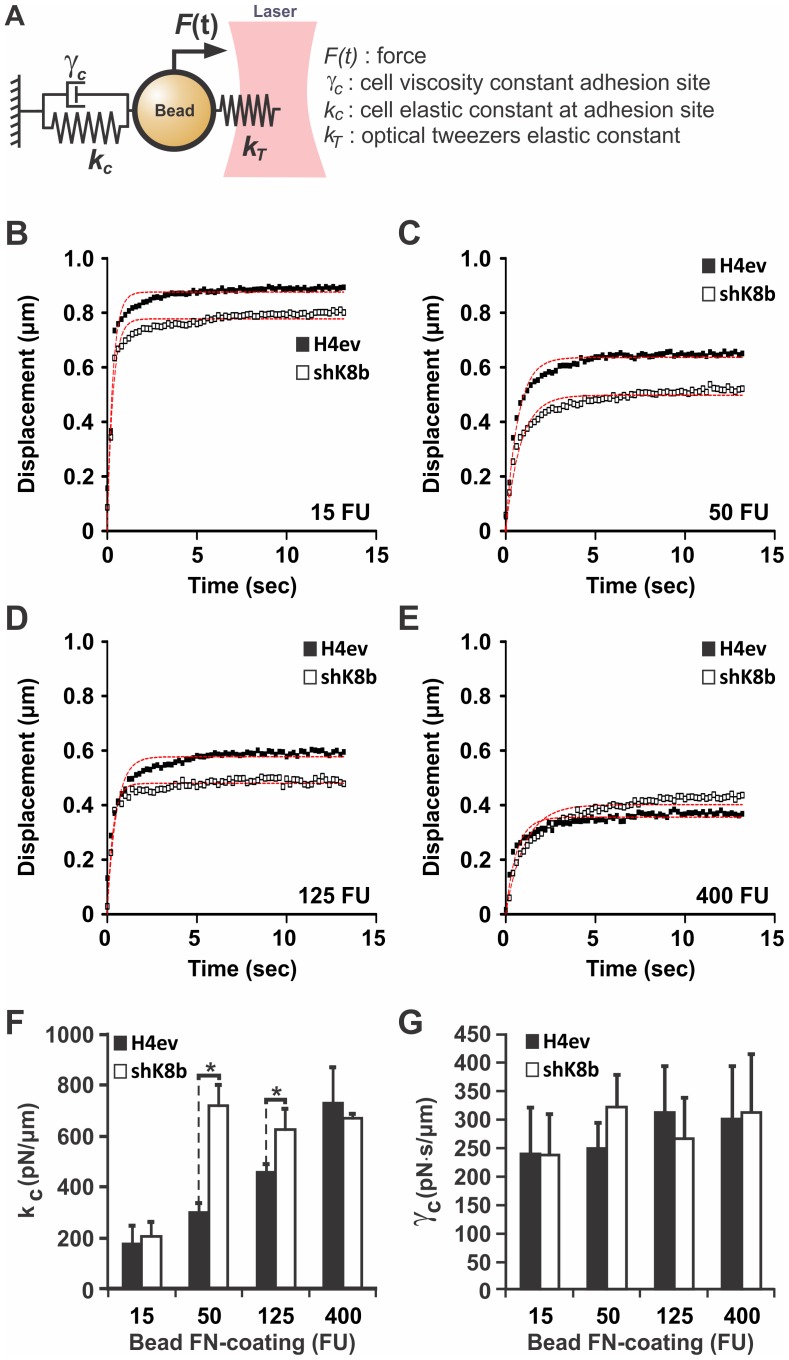
H4ev and shK8b cell stiffness as function of bead FN-coating density. (A) Biomechanical model used to evaluate cell elastic and viscous parameters as function of the optical tweezers elastic constant and initial force. H4ev versus shK8b cells are seeded in FN-coated glass bottom dishes, which constitute a very high rigidity substrate (>3 GPa), and are allowed to form monolayers. Thereafter, beads exhibiting a FN coating of (B) 15, (C) 50, (D) 125 and (E) 400 fluorescent units (FU) are allowed to attach for 1 hr on the monolayers, and their displacements measured with the optical tweezers. Average displacement curves are generated from 40 independent bead measurements. The dotted curves present in each graph correspond to the numerical fit obtained from our mechanical model. The cell (F) elastic constant k_c_ and (G) viscosity constant γ_c_ are computed according to the model and the average is obtained from 3 separate experiments. Bars denote SE. *, p<0.05 for H4ev versus shK8b.

The cell elasticity at FAs, created by the binding of 3–5 µm FN-coated beads, has been assessed before [7], [8], based on the cell mechanics model described by Fung [32]. Here, we added a spring, representing the optical tweezers, to the Voigt body comprised of a spring in parallel with a dashpot as originally proposed in Fung's model (Fig. 3A). Since the optical tweezers-generated force *F(t)* is a function of the bead displacement *ε(t)*, the force can be expressed as:
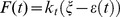
(1)where the initial force applied to the bead depends on *k_t_*, the trap elastic constant, and ξ, the distance of the bead from the trap center (at *t* = 0). We can then write the differential equation for a Voigt body by substituting the force with Eq.(1):
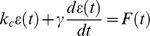


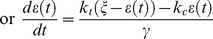
(2)which introduces the cell local elastic parameter *k_c_* and viscous parameter *γ_c_* (Fig. 3A). The corresponding solution for the bead displacement *ε(t)* is given by:
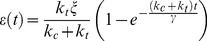
(3)The local cell parameters *k_c_* and *γ_c_*, are numerically extracted from Eq. (3), using a least square fit on the experimental data measured for the bead displacement *ε(t)*.

In such a model, the FN-coated bead becomes a mechanical linker between the optical tweezers and the cell. Interestingly, the force needed to break this mechanical link has been shown by others to be influenced by the FN density on the bead surface [33], [34]. We thus assessed to which extend the K8/K18 modulation of the local cell mechanical parameters *k_c_* and *γ_c_* at FAs was affected by the bead FN-coating density. Accordingly, flow cytometry was used to quantify the bead FN coating, in terms of relative fluorescence units (FUs) after FN immunolabeling. Thereafter, beads exhibiting relevant FN-coating densities, namely 4 sets of beads exhibiting 15, 50, 125 and 400 FUs respectively, were selected (Fig. S1). As shown in Fig. 3B–E, the average bead displacement *ε(t)*, derived from one experiment of 40 independent measurements using each set of FN-coated beads, was evaluated on both H4ev and shK8b cells seeded on FN-coated glass bottom dishes. A least-square fit was performed for each average displacement *ε(t)*. For a low FN-coating density (15 FUs), the measured average displacement *ε(t)* of the FN-coated bead was similar for both H4ev and shK8b cells (Fig. 3B). In the case of intermediate FN densities (beads with 50 and 125 FUs), shK8b cells exhibited a decreased FN-coated bead displacement *ε(t)* when compared to H4ev (Fig. 3C–D). At the highest amount of FN-coating (400 FU), the bead displacement was similar for both the H4ev cells and the shK8b cells (Fig. 3E). Analysis of the computed parameters from three sets of experiments, i.e. a total of 120 beads for each FN-coated bead condition, revealed that the elastic compliance *k_c_* follows a steady increase in H4ev cells, relative to FN-coating density on the beads (Fig. 3F). In contrast, the elasticity *k_c_* of shK8b cells increased nearly 4-fold to reach its maximum, when using 50 FU FN-coated beads instead of 15 FU FN-coated beads. In contrast, we found no difference for the viscous parameter *γ_c_* between H4ev and shK8b cells over the FN-coated bead coating range (Fig. 3G). On these grounds, it appears that a K8/K18 IF loss in hepatic cells results in a firmer mechanical coupling between the bead and cell FAs, which reaches saturation at intermediate FN-coating density.

**Figure 4:**
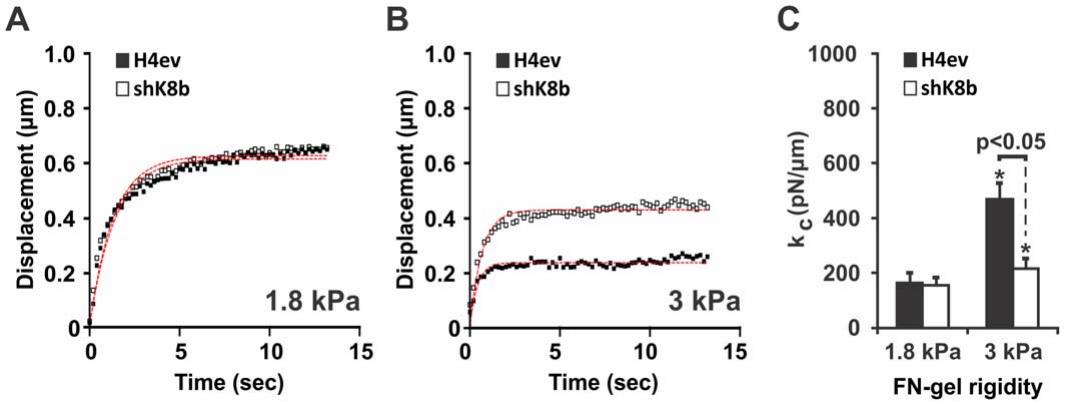
H4ev and shK8b cell stiffness as function of FN-gel rigidity. Mean bead displacement curves of one data set containing 40 independent bead (400 FU beads) measurements for cells plated on (A) 1.8 kPa gel and (B) 3 kPa gel for both H4ev and shK8b cells, along with the numerical fits (dotted line). (C) The corresponding 3- separate experiment averages of the computed elastic constant k_c_, showing a differential stiffness increase from 1.8 kPa to 3 kPa in H4ev versus shK8b cells. *, p<0.05 relative to 1.8 kPa gels.

In light of the actin fiber organization modulation we observed over the different FN-gel rigidities (Fig. 1), we determined whether a correlation was taking place between actin fiber organization and cell stiffness at the bead adhesion site. To eliminate a potential difference related to bead mechanical coupling, we selected here the 400 FU FN-coated beads, based on the finding showing equivalent local stiffness for both H4ev and shK8b cell seeded on FN-coated glass (see Fig. 3F). As seen in Fig. 4A, H4ev and shK8b cells seeded on 1.8 kPa FN-gels yielded comparable induced FN-coated bead displacements. However, when the FN-gel rigidity was increased to 3 kPa, the average FN-coated bead displacement was significantly reduced on H4ev cells, and to a lower extent on shK8b cells (Fig. 4B). As a result, the computed cell elastic constant on a 3 kPa FN-gel was significantly lower for shK8b cells than for H4ev cells (Fig. 4C). Thus, hepatic cells lacking K8/K18 IFs are unable to adequately match their stiffness with the rigidity of the underlying FN-gel substratum.

### K8/K18 IF modulation of hepatic cell stiffness is Rho-ROCK dependent

**Figure 5:**
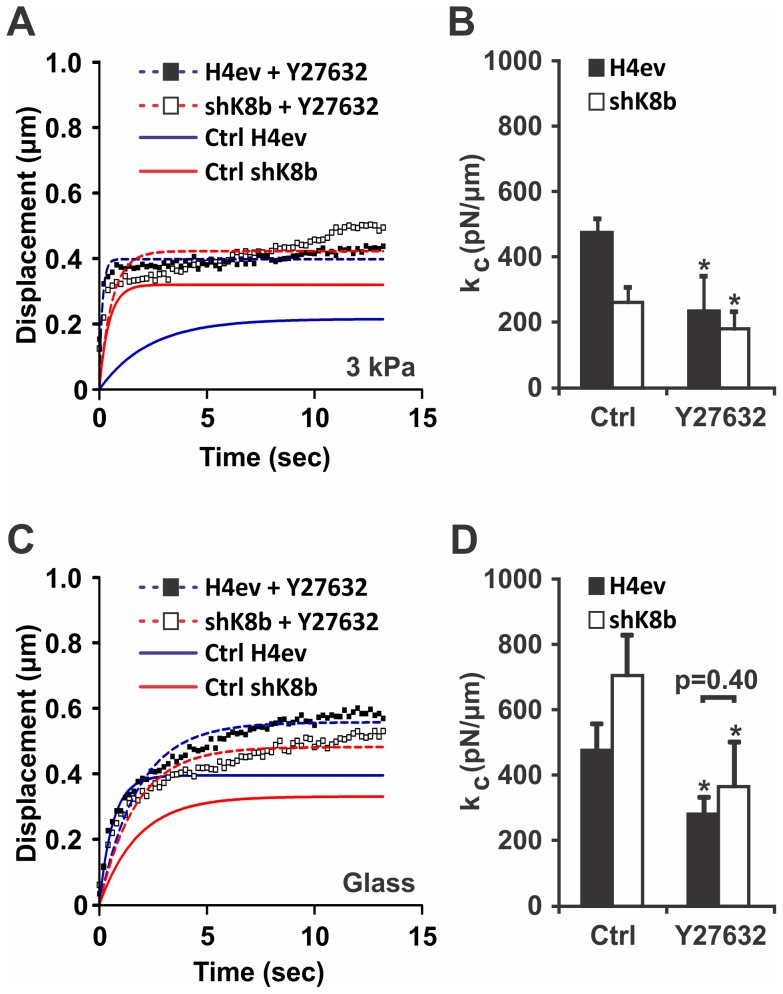
ROCK involvement in K8/K18 IF modulation of cell stiffness. (A) Mean bead displacement curves of one data set containing for 400 FU beads attached to a monolayer of cells seeded on a 3 kPa gel, following addition of Y27632 (1 µM, 30 min). (B) The corresponding cell elastic constant k_c_ obtained in presence of vehicle (Ctrl) or Y27632. The k_c_ difference between H4ev and shK8b cell treated with Y27632 is not statistically significant (p = 0.26) (C) Mean bead displacement curves of one data set containing for 125 FU beads attached to a monolayer of cells seeded in a FN-coated dish, following addition of Y27632. (D) The corresponding cell elastic constant k_c_ obtained in absence (Ctrl) or presence of Y27632. The k_c_ difference between H4ev and shK8b cell treated with Y27632 is not statistically significant (p = 0.40). The dotted lines correspond to the numerical fits on Y27632-treated cells, while the solid lines correspond to the numerical fit on control cells data. Bars denote SE. *, p<0.05 relative to controls.

The Rho-ROCK pathway is involved in cell adaptation to substratum rigidity by controlling cell stiffness [27]. We thus assessed whether Rho-ROCK was involved in the differential H4ev versus shK8b cell stiffness measured at FAs. As shown in Fig. 5A, a treatment with the ROCK inhibitor Y27632 increased the displacement of 400 FU FN-coated beads attached to either H4ev or shK8b cells cultured on the 3 kPa FN-gel. Intriguingly, following ROCK inhibition in shK8b cells, the beads maintained a discernible creeping motion, which resulted to a slight distortion of the fitting curve. Nevertheless, it remained that the calculated stiffness values were not statistically different between for both Y27632-treated H4ev and shK8b cells (Fig. 5B). Moreover, measurements with 125 FU FN-coated beads attached to either H4ev or shK8b cells seeded on FN-coated glass yielded equivalent bead displacements following Y27632 treatment, when compared to control untreated cells (Fig. 5C). In the same way, the stiffness difference computed for untreated cells was abolished after ROCK inhibition (Fig. 5D). Together, the results indicate that the K8/K18 differential modulation of cell stiffness at FAs is ROCK dependent.

### K8/K18 IF loss perturbs Rho regulation of actin dynamics

**Figure 6:**
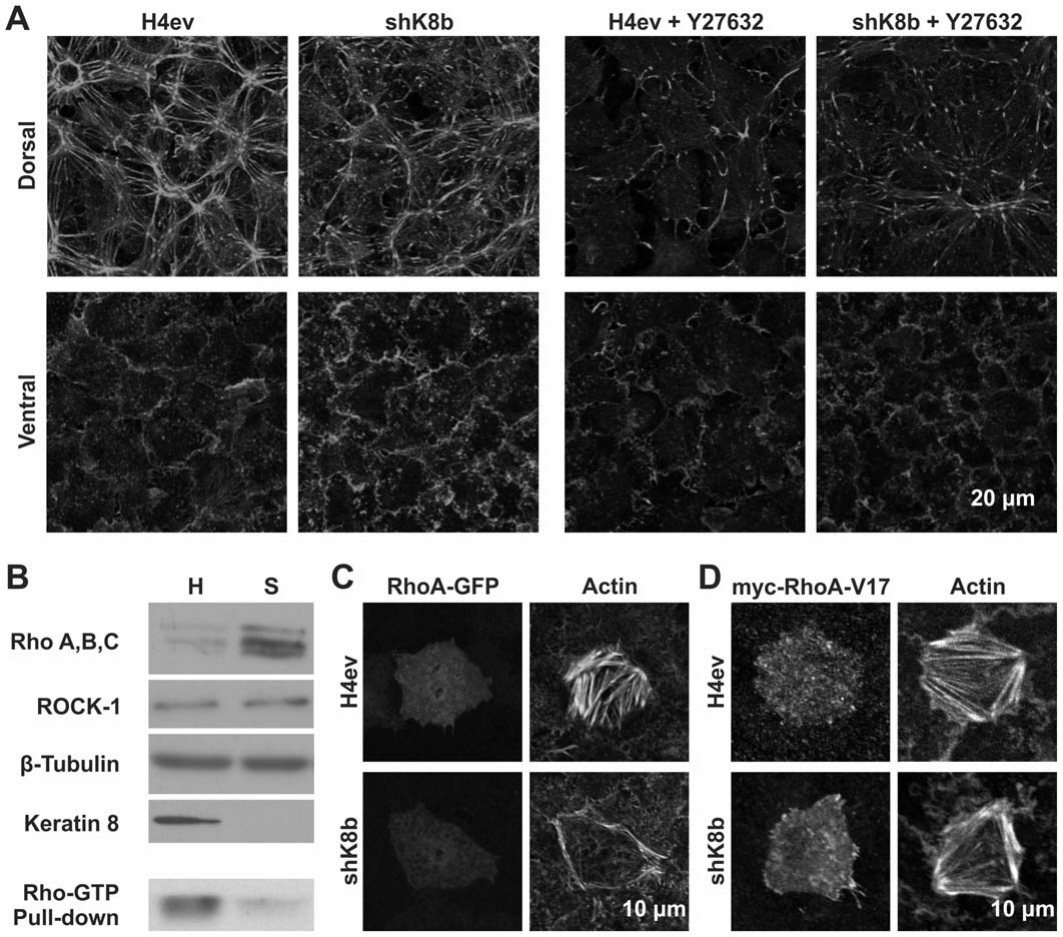
K8/K18 IF modulation of Rho-mediated actin fiber organization. (A) Confocal images of fibrillar actin at dorsal and ventral cell surfaces, following addition of Y27632 (1 µM, 1 h) on 3-day monolayers serum-starved overnight, showing that ROCK inhibition disrupts H4ev cell actin fiber organization at the basal and apical surface membranes to a greater extent in H4ev cells than in shK8b cells compare to untreated cells. (B) Western blottings of total Rho (A, B, C) and ROCK-1 showing increased Rho level in shK8b versus H4ev cells; Rho-GTP pull-down assay, showing a higher Rho activation in H4ev versus shK8b cells, despite a lower Rho (A, B, C) content. Confocal images of fibrillar actin at the ventral cell surface, showing cells expressing either a (C) RhoA-GFP or (D) a constitutively active mutant myc-RhoA-V17; RhoA-GFP expression induces the formation of dense actin fibers only in H4ev cells, while myc-RhoA-V17 induces the formation of comparable actin fibers in both H4ev and shK8b cells.

The RhoA-ROCK signaling pathway is a key regulator of cell stiffness through a control of actin contractility and fiber organization [1], [6]. In this context, we addressed the K8/K18 IF involvement in the Rho-mediated regulation of actin dynamics. H4ev and shK8b cells were seeded on FN-coated glass and the actin fiber organization in absence or presence of ROCK Y27632 inhibitor was assessed. In absence of the inhibitor, the actin fiber distribution was profoundly re-arranged at the cell dorsal surface in shK8b versus H4ev cells, but less affected at their ventral surface (Fig. 6A). While its presence led to a significant overall disruption of the actin fibers in both H4ev and shK8b cells, this ROCK inhibition did not result in a complete disassembly of the actin fibers at the dorsal surface of shK8b cells.

Given these results, we assessed Rho expression and activity in H4ev versus shK8b cells. As shown in Fig. 6B, the expression levels of RhoA, B and C were higher in shK8b than in H4ev cells. Of note, the level of ROCK remained the same for both cell types. Using a pull-down assay based on the GTP-bound Rho specific interaction with the Rotekin Rho-binding domain, we determined whether the higher protein level found in shK8b cells was translatable into increase Rho activity. The results showed that in absence of K8/K18 IFs, the amount of Rho-GTP was greatly diminished (Fig. 6B), thus suggesting that Rho activation was impaired in shK8b cells.

To further document this possibility, we overexpressed a GFP-tagged version of RhoA in both H4ev and shK8b cells, and then imaged the actin fiber organization. In line with the above data, RhoA-GFP expression led to the formation of a prominent actin fiber network in H4ev cells, but not in shK8b cells (Fig. 6C). Still, some actin fibers appeared at the periphery of RhoA-expressing shK8b cells, indicating that RhoA overexpression had some regional effect on actin polymerization, in spite of the K8/K18 IF loss, indicating that K8/K18 IFs modulate the localization-dependent activity of RhoA. In addition, the expression of a constitutively active mutant of RhoA (RhoA-V17) led to the formation of actin fibers exhibiting a similar organization in both H4ev and shK8b cells (Fig. 6D), strongly supporting a K8/K18 IF effect on Rho activation. Together with the above data on cell mechanics, the present results establish that the Rho-ROCK pathway is perturbed in absence of K8/K18 IFs, which in turn leads to impaired actin cytoskeleton dynamics, and cell stiffness.

## Discussion

The work reported here uncovers a key molecular mechanism for the involvement of K8/K18 IFs as modulators of hepatic cell mechanics. By comparing shK8b versus H4ev cell adaptation to ECM-derived mechanical cues, using FN-gels of controlled rigidity and measuring cell stiffness with an optical tweezers, we find that a K8/K18 IF loss perturbs the mechanotransduction by: altering the cell mechanosensing, along with a marked actin fiber differential re-organization; interfering with the proper match between cell stiffness and ECM rigidity; and impairing the Rho-ROCK pathway controlling cell stiffness and actin dynamics. Overall, the results reveal a K8/K18 IF contribution to the cell stiffness-ECM mechanosensing interplay through a modulation of Rho-dependent actin cytoskeleton dynamics in simple epithelial cells.

Substratum rigidity has a fundamental impact on molecular events triggered at FAs/integrins, which in turn modulate cell spreading and actin cytoskeleton organization [1]. Notably, the range of rigidity where this process can be observed is cell-type dependent. For instance, fibroblasts achieve a maximal spreading and display prominent actin fibers on a substratum of 10 kPa [27], while chondrocytes still maintain a round shape at this rigidity [35]. In contrast, mammary epithelial cells undergo a round to spread shape transition between 0.4 and 5 kPa, while actin fibers are detected only above 5 kPa [5]. In comparison, MDCK cells achieve a spread shape at rigidity of 1 kPa [36]. As expected, the present results indicate that the round to spread shape transition in H4ev cells occurs at rigidity level within the range for typical simple epithelial cells [5], [36]. In addition, previous work using fibroblasts and endothelial cells has indicated that the influence of substratum rigidity on cell spreading (shape) and cytoskeletal organization can be modulated by cell-cell contacts, particularly in the case of endothelial cells [27]. Conversely, other line of work using the latter cell type has revealed that the rigidity of a substratum adhering on FAs/integrins can affect cell-cell (e.g. actin-associated) junction stability, through a modulation of the Rho-mediated actin cytoskeleton contractility [37]. The cells of interest here, derived from simple epithelium, contain not only actin-linked adherens junctions but also keratin-associated desmosomal junctions, which allow formation of a continuous K8/K18 IF network across the cell monolayer. In this context, one can assume that any change in hepatic cell shape (spreading), as result of the K8/K18 IF loss in shK8b cells, is possibly due to an effect on both substratum rigidity and cell-cell contacts. However, the present results show that shK8b cells fail to initiate cell spreading at the lowest rigidity, regardless of cell-cell contacts, where H4ev cells are able to do so, indicating a predominant K8/K18 IFs involvement at events triggered at FAs/integrins rather that at cell-cell contacts at this rigidity level. Moreover, the shape transition in shK8b cells takes place at a higher rigidity threshold, thus providing the first indication that K8/K18 IFs behave as modulators of the hepatic cell mechanosensing of substratum rigidity. In addition, it appears that K8/K18 IFs constitute a modulator of actin fiber organization over a wide range of ECM rigidity, suggesting that the K8/K18 IF-dependent mechanosensing at FAs/integrins is primarily mediated through actin cytoskeleton. In this context, the likely involvement of cell lineage specific-IFs as key regulators in the setting of the actin cytoskeleton-dependent mechanosensing range in simple epithelial cells constitutes an appealing issue.

Stiffness measurements on cells seeded on ECM substrata of various rigidities have been performed using different force detecting systems, including magnetic twisting cytometry, atomic force microscopy and optical tweezers [38], [39], [40]. Of particular note, both fibroblasts and mesenchymal stem cells have been found to possess a stiffening behavior that depends on substratum rigidity [38], [39]. In line with these findings, our present data point to a stiffening behavior in both H4ev and shK8b epithelial cells, with the difference that hepatic cells lacking K8/K18 IFs stiffen on higher gel rigidity, in correlation with the increased rigidity required to form a cell monolayer. Of note, measurements of alveolar epithelial cell stiffness did not reveal a ECM rigidity-dependent sensitivity [40]; however, these cell measurements have been made at ECM rigidity levels well above the normal lung tissue rigidity [1]. Actually, there is a saturation point for cell stiffness above a certain level of ECM rigidity [38], stressing the importance of addressing the relationship between ECM rigidity and cell mechanics within physiologically relevant experimental settings. Moreover, the differential actin fiber organization we observe here in hepatic epithelial cells seeded on glass substratum does not correlate directly with the stiffness measurements. Furthermore, both bead size and ECM-coating density have been shown to influence bead adhesion and the associated integrin-mediated mechanical coupling [41]. In this context, the stiffness differences observed here in shK8b cells versus H4ev cells upon variation of the bead FN-coating density are likely due to an increased integrin-mediated bead-actin cytoskeleton coupling. Overall, we conclude that the K8/K18 IF loss perturbs the shK8b cell ability to respond to changes in both substratum rigidity and ECM-bead coating density characterized by an altered ability to tune their own stiffness.

There is accumulating evidence indicating that substratum rigidity influences cell stiffness through actomyosin mediation [39], [42]. Notably, the control of actomyosin contractility and actin fiber formation is known to be dependent on the Rho-ROCK signaling pathway, which in turn activates different effectors of actin organization [5], [12]. Although RhoA is the most characterized among the Rho family members, both RhoA and RhoC can induce formation of fibrillar actin through ROCK activation [43]. In addition, the activation of Rho proteins is compartmentalized, meaning that different pools of the same Rho protein can be associated with distinctive actin organization traits [44]. The present results revealing a differential actin fiber distribution at the dorsal surface of H4ev versus shK8b cells, and a peripheral localization of actin fibers at the ventral surface of shK8b cells following RhoA overexpression, are consistent with such a Rho compartmentalization. Still, the persistence of some fibrillar actin at the dorsal surface in shK8b cells following ROCK inhibition suggests that alternate signaling pathway(s) can contribute upon a RhoA impairment, in line with previous data revealing a regional fibrillar actin formation through myosin light chain kinase [45]. Nevertheless, by combining these findings with those on cell stiffness, it remains that K8/K18 IFs are involved in actin cytoskeleton organization and hepatic cell mechanics, through a strategic modulation of RhoA-ROCK activation.

The present results reveal an altered bead-cytoskeleton coupling and mechanosensing triggered by substratum rigidity at FAs/integrins. Notably, integrin engagement on their respective substratum can regulate Rho activity through the involvement of Src family kinases. Indeed, Src controls RhoA activity by regulating a number of guanine-exchange factors (GEF) and GTPase-activating proteins (GAP), including p190RhoGAP and p190RhoGEF [46]. During initial cell spreading, Src enhances p190RhoGAP phosphorylation, leading to a down-regulation of RhoA activity [47], whereas at a later spreading stage, Src can increase RhoA activity through p190RhoGEF and promote actin fiber formation [48]. Of additional note, Src activation has been linked to alterations of local cell stiffness at FAs, as measured by FN- or vitronectin-coated bead pulling [6], [41]. On this ground, a K8/K18 IF modulation of the Src-mediated bead-mechanical coupling at FAs constitutes an appealing perspective.

We have shown a few years ago that much of the K8/K18 IFs and fibrillar actin are distributed beneath the surface membrane in hepatic cells [24], [26], [49]; we have also found a similar distribution for plectin, a cytolinker for IF proteins, actin and microtubule-associated proteins [50]. More recently, we have reported that K8/K18 IFs in hepatic cells modulate the sub-cellular localization/interaction of plectin, PKC (“protein kinase C”) and RACK1 (“Receptor of activated C kinase”), in link with the integrin-dependent adhesion and migration [22]. In the work reported here, using the same cell model, it appears that K8/K18 IFs are modulators of the RhoA localization-dependent activity. Since RACK1 also comprises a binding site for Src [22], [51] and since Src is a major regulator of Rho activation [46], we propose that the interplay between K8/K18 IFs and Rho-mediated actin dynamics occurs through a plectin-RACK1-Src connection at FAs in hepatic cells.

In addition, considering the relation between K8 point mutations and the predisposition to liver cirrhosis [19], and the link between cirrhosis and hepatocarcinogenesis [21], hepatic cells offer a model of choice to address the role of K8/K18 IFs as modulators of the mechanotransduction taking place during the cell tumorigenic process in simple epithelia. Finally, since K8 and K18 constitute the first cytoplasmic IF genes expressed in the embryo [16] and since mechanical force plays a key role in governing stem cell differentiation [1], the possibility that K8/K18 IFs intervene in the mechanical stimulation of stem cell lineage emergence during early development constitutes an attractive perspective.

## Materials and Methods

### Reagents

Primary antibodies used were: mouse monoclonal anti-Rho (A, B, C) (#05-778; Millipore, Mississauga, ON, Canada); mouse monoclonal anti-ROCK1 (#611136; BD Pharmingen, Mississauga, ON, Canada); rabbit polyclonal anti-human fibronectin (FN) (#F3648), mouse monoclonal anti-Tubulin (#T5293) and mouse monoclonal anti-FLAG M2 (#F1804; Sigma-Aldrich Canada Ltd, Mississauga, ON, Canada); and mouse monoclonal anti-rat K8 [52]. Secondary antibodies used were: Alexa Fluor® 488-goat anti-mouse IgG, Alexa Fluor® 555-goat anti-mouse IgG and Alexa Fluor® 594-goat anti-rabbit IgG (Invitrogen, Burlington, ON, Canada); Horseradish peroxidase–goat anti-rabbit IgG and anti-mouse IgG (Bio-Can Inc, Mississauga, ON, Canada). Human FN was purified as described before [24], [53]. Fetal Bovine Serum (FBS) was purchased from Wisent Inc (Quebec, Qc, Canada). GFP-RhoA plasmid was kindly provided from Dr. Josée N. Lavoie (CRCHUQ, Quebec, Ca) [54], and Myc-RhoA V17 plasmid from Dr Nathalie Lamarche-Vane (McGill University, Montreal, Ca) [55]. The Rho activation assay kit was purchased from Millipore (#17-294, Mississauga, ON, Canada). Y27632 ROCK inhibitor was purchased from Sigma (#Y0503, Sigma-Aldrich Canada Ltd). Sulfo-SANPAH was purchased from Fisher (#22589, Fisher Canada, Ottawa, ON, Canada). All other chemicals were from Sigma-Aldrich Canada Ltd.

### Fibronectin-coated polyacrylamide gel preparation

FN-coated polyacrylamide gel (FN-gel) was prepared according to the Wang and Pelham's procedure [25], with the following specifications: the gel rigidity was controlled by varying bis-acrylamide ratio (0.03% to 0.08%) for a fixed acrylamide amount (5%); a 10 µl drop of polyacrylamide was put on the functionalized glass surface (glass bottom culture dish, WPI, Sarasota, Fl, USA), and the drop was flattened to approximately 40 µm thick using a 18 mm cover glass; and gels were coated overnight at 4°C with FN (10 µg/ml) after using a photoactivatable sulfonated cross-linker. The Young modulus was measured from the macroscopic deformation of gels slab (4 mm×2 cm×7 cm) put under constant strain.

### Stable K8-knockdown and empty vector H4-II-E-C3 cells

Stable K8-knockdown (shK8b) and empty vector (H4ev) H4-II-E-C3 cells, generated by the shRNA technique, have been described previously [24]. Cells were maintained in DMEM, supplemented with 10% FBS and 100 µg/ml streptomycin, at 37°C in a humidified atmosphere of 5% CO_2_/95% air. The shK8b1 cells are routinely monitored for the K8 loss, as described before [24]. Cells were seeded at an intermediate density of 3.5×10^4^ cells/cm^2^ in culture glass bottom dishes either pre-coated with 10 µg/ml FN [9], [26] or coupled to FN-gels. At this density, both single cells and cells exhibiting cell-cell contacts were present at 24 hr post-seeding. Measurements of cell area were performed, as before [24], on both single cells and cells exhibiting cell-cell contacts. Transfections were performed with the Fu-Gene HD® transfection Reagent (#E2311; Promega, Madison, WI, USA), according to the manufacturer's instructions.

### Bead preparation

FN-coated beads were prepared according to the manufacturer's protocol (Banglabs, Fishers, IN, USA) with the following specifications: The reaction buffer pH ranged from 5.5 to 7.5, in order to modulate the coupling efficiencies of FN to the beads. Briefly, 5 µm polystyrene beads were washed twice in the reaction buffer, incubated 15 min in presence of carbodiimide, and washed 3 times. Beads were then subjected to a 4 h-incubation in presence of 100 µg FN. FN-coated bead aggregation was remedied by two short 7 sec sonication bursts at the end of the coupling procedure. Beads were further blocked with BSA for 1 h. In some experiments, FN-coated beads were incubated overnight at 4°C with anti-FN followed with an Alexa Fluor® 488 anti-mouse at room temperature (RT) for 1 h for further analysis of bead FN coating by flow cytometry (EPICS Xl MCL, Beckman Coulter, Mississauga, ON, Canada). Negative controls were made with beads that were subjected only to the blocking procedure.

### Optical tweezers experimental procedure

Setup of the optical system was as reported previously [26], except that a 5 mW HeNe laser was introduced for a continuous tracking of the bead position (Fig. S2). Calibration of the optical tweezers trapping force was performed as before [9]. This procedure was used for glass bottom dishes coated with FN only or covered with a 40 µm-thick FN-gel. The calculated optical tweezers elastic constant in cover glass dishes coated with FN was 522±25 pN/µm, and 221±52 pN/µm in glass bottom dish with the FN-gel.

Approximately 10000 FN-coated beads were added to a cell monolayer 1 h before each bead displacement measurement. Prior to force application, the bead was positioned with the piezoelectric stage at 0.9±0.1 µm away from the trap center along a single axis by automated piezoelectric positioning. For each assessment, the bead displacement signal was acquired during a 15-sec timeframe on a bead-by-bead basis, for 40 beads bound at the dorsal surface of single cells within the same monolayer [9], [26]. Here, the laser tweezers shutter was synchronized with the acquisition to open with a 1 sec delay.

### Western blotting

Total proteins were extracted with preheated (90°C) 2X sample buffer [56]. Twenty µg of protein extract were subjected to sodium dodecyl sulfate (SDS)-polyacrylamide gel electrophoresis and electro-transferred onto a polyvinylidene difluoride membrane. The blots were incubated with the primary antibody for 16 h at 4°C and then with the appropriate horseradish peroxidase-conjugated secondary antibody for 1 h at RT. The peroxidase signal was revealed with the SuperSignal West Pico kit (#34077, Fisher Canada, Ottawa, ON, Canada) or the Lumi-LightPlus Western Blotting Substrate (#12015196001, Roche, Laval, Qc, Canada).

### Rho activation assay

GTP-bound active Rho was assessed by pull-down assays following the manufacturer's instructions (Millipore). Briefly, H4ev and shK8b cells were cultured to approximately 90% confluence before being subjected to a 24 h serum starvation. Cell lysate was incubated together with beads coupled to the Rhotekin Rho binding domain. Precipitated GTP-bound Rho was subjected to the western blot procedure described above, using 12% SDS-PAGE and immunoblotting with the anti-Rho (A, B, C) monoclonal antibody.

### Confocal cell imaging

Cells were washed twice with PBS, fixed for 10 min at RT with 3.7% formaldehyde in PBS, followed by methanol∶acetone (3∶7) for 10 min at −20°C. They were then incubated successively, overnight at 4°C with anti-Flag M2 (Myc Flag) (1/50 in PBS) or anti-GFP followed with Alexa Fluor® 488 anti-rabbit IgG antibody at RT for 1 h and then with phalloidin-Alexa Fluor® 594 (1/40 in PBS) at RT for 1 h. Images were captured with a FV1000 confocal system (Olympus Canada), using the 488 and 543 nm excitation laser lines and either a 20×/0.45 NA objective or a 100×/1.40 NA oil immersion objective. Image maximum projection and optical section slice were performed with the ImageJ software (NIH Bethesda, MD, USA). Evaluations of the actin fiber lengths were performed on three 20× confocal image (317.6 µm×317.6 µm) projections from three independent experiments, using the tubeness plugin in the ImageJ software. A threshold was applied on the resulting images, prior to actin fragment length measurements.

### Data processing and statistical analysis

Mathematical processing of the experimental data to compute the *k_c_* parameter was performed initially with the Matlab software (The Mathworks, Natick, Massachusetts, USA). In addition, the bead displacement curves and numerical fit overlays were plotted with the Prism software (GraphPad Software, La Jolla, CA, USA) and then, the numerical fits were used to extract the *γ_c_* parameter and the *k_c_* parameter, as well. Two groups of data were compared by a *t test* statistical analysis, using the Matlab software. A p value below 0.05 was considered statistically significant.

## Supporting Information

Figure S1 **Flow cytometry quantifications of beads exhibiting different FN-coating densities, in terms relative fluorescence units (FUs), following FN immunolabeling. Control corresponds to albumin-coated beads (Ctrl).**
(TIF)

Figure S2 **Optical tweezers experimental setup.** (A) The optical tweezers operate around a linearly polarized 5W ND:YVO4 fiber laser (IPG Photonics, Oxford, MA, USA) emitting at 1070 nm coupled in the back aperture of a 100×/1.3NA oil immersion objective (Olympus Canada, Mississauga, ON, Canada). The 5 mW HeNe laser is always operational and used for tracking bead position through a position sensitive photodiode (PSD, S1880 Hamamatsu, Bridgewater, NJ, USA); M1 to M5 are gimbal mounted dichroic mirrors used for beam steering. The lens L1 images the sample upon the CCD camera and lens L2–L3 act as a telescope to adjust the detection laser width. A beam sampler plate BS redirects backscattered light from the sample to the PSD to monitor bead position. A Gland prism (GP) and a λ/2 wave plate are used for attenuation of the trapping laser intensity. (B) Pictogram of an optical tweezers sample zoomed view depicting the FN-coated bead adhesion to the cell dorsal surface.
(TIF)
