# Supplementary figures and images for: Keratin 8/18 Regulation of Cell Stiffness-Extracellular Matrix Interplay through Modulation of Rho-Mediated Actin Cytoskeleton Dynamics
Source: PLoS One. 2012 Jun 7;7(6):e38780. doi: 10.1371/journal.pone.0038780 (PMC3369864; doi:10.1371/journal.pone.0038780)

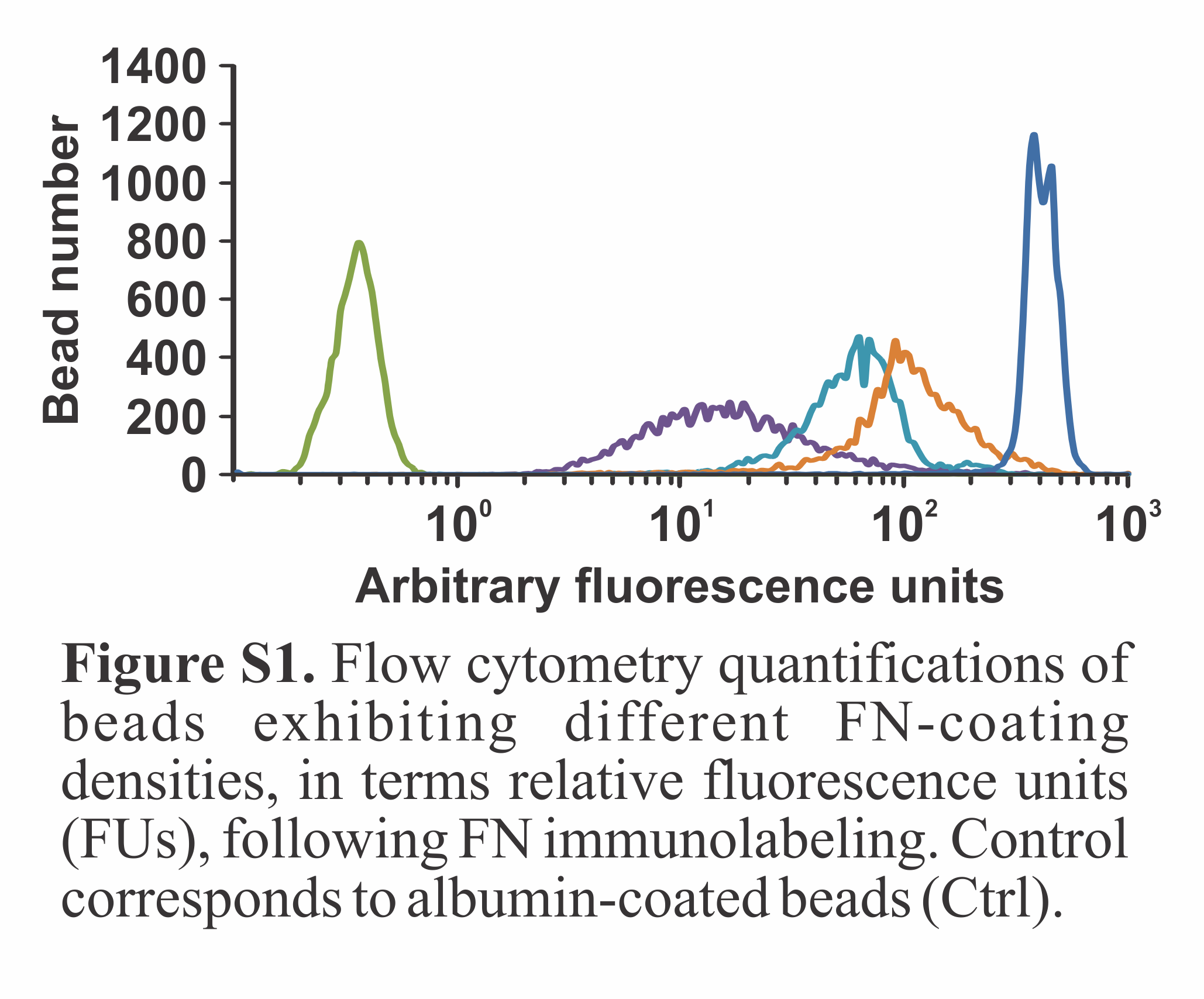

Supplement: Figure S1 — Flow cytometry quantifications of beads exhibiting different FN-coating densities, in terms relative fluorescence units (FUs), following FN immunolabeling. Control corresponds to albumin-coated beads (Ctrl). (TIF) [file pone.0038780.s001.tif]

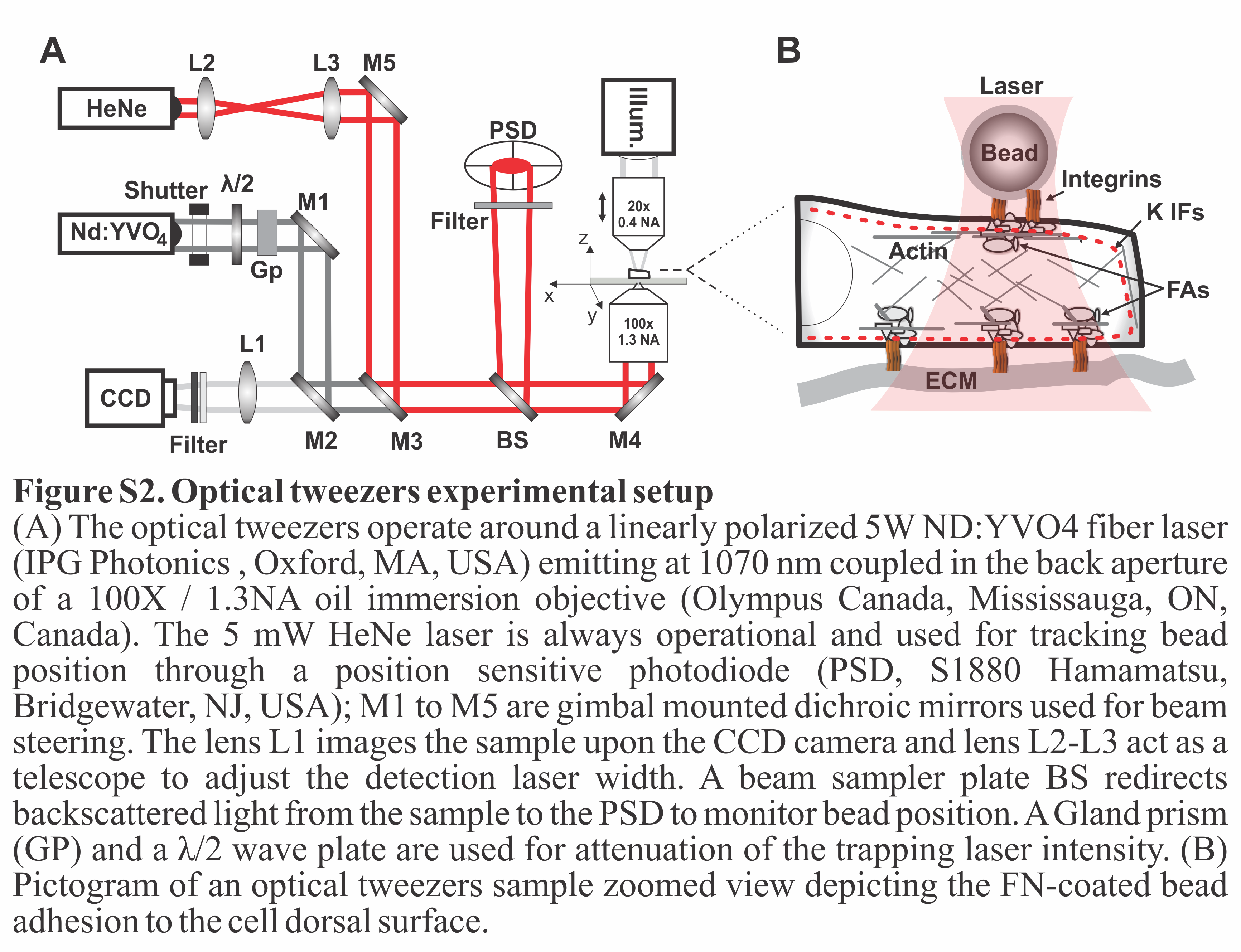

Supplement: Figure S2 — Optical tweezers experimental setup. (A) The optical tweezers operate around a linearly polarized 5W ND:YVO4 fiber laser (IPG Photonics, Oxford, MA, USA) emitting at 1070 nm coupled in the back aperture of a 100×/1.3NA oil immersion objective (Olympus Canada, Mississauga, ON, Canada). The 5 mW HeNe laser is always operational and used for tracking bead position through a position sensitive photodiode (PSD, S1880 Hamamatsu, Bridgewater, NJ, USA); M1 to M5 are gimbal mounted dichroic mirrors used for beam steering. The lens L1 images the sample upon the CCD camera and lens L2–L3 act as a telescope to adjust the detection laser width. A beam sampler plate BS redirects backscattered light from the sample to the PSD to monitor bead position. A Gland prism (GP) and a λ/2 wave plate are used for attenuation of the trapping laser intensity. (B) Pictogram of an optical tweezers sample zoomed view depicting the FN-coated bead adhesion to the cell dorsal surface. (TIF) [file pone.0038780.s002.tif]
